# Mpox Outbreak in the East Africa Region: Current Status, Containment Measures, Challenges, and Future Direction

**DOI:** 10.1002/puh2.70194

**Published:** 2026-02-09

**Authors:** Reuben Sindayiheba, Noel Gahamanyi, Jerome Ndayisenga, Edouard Ntagwabira, Emmanuel Nsengiyumva, Emmanuel Kabalisa, Gashegu Misbah, Isabelle Mukagatare, Leon Mutesa, Claude Mambo Muvunyi

**Affiliations:** ^1^ Rwanda Biomedical Centre Kigali Rwanda; ^2^ School of Health Sciences, College of Medicine and Health Sciences University of Rwanda Kigali Rwanda; ^3^ Center for Human Genetics, School of Medicine and Pharmacy, College of Medicine and Health Sciences University of Rwanda Kigali Rwanda

**Keywords:** East African Community (EAC), Mpox, outbreak response, public health surveillance, vaccination

## Abstract

In 2024, the World Health Organization declared Mpox outbreak a Public Health Emergency of International Concern. The epicenter of this outbreak was the Democratic Republic of Congo (DRC), which is a member of the East African Community (EAC). The majority of EAC countries reported Mpox cases and associated deaths. Mpox outbreak negatively affected EAC member states but triggered the strengthening of diagnostic and surveillance capacity. This review assessed the status, containment measures, challenges, and future directions of Mpox in the EAC. As of July 27, 2025, the DRC was the top country in the world with Mpox with 28,165 cases and 69 deaths, followed by Uganda, Burundi, Kenya, Rwanda, Tanzania, and South Sudan with 7648 cases and 48 deaths, 4231 cases and one death, 281 cases and five deaths, 124 cases, 111 cases, and 17 cases, respectively. Rwanda, Tanzania, and South Sudan have not recorded any death associated with Mpox. The DRC, Uganda, and Burundi experienced extensive transmission rates, whereas Kenya and Uganda had higher case fatality rates of 1.78 and 0.63, respectively. Containment measures in EAC countries included enhanced surveillance, public awareness campaigns, training on infection prevention and control, and vaccination. Of the 801,000 Modified Vaccinia Ankara‐Bavarian Nordic (MVA‐BN) vaccine doses administered, over 73% were administered in the DRC. Challenges, including inadequate healthcare infrastructure and misinformation, affected Mpox control measures. This study underlines the need for a joint regional strategy to strengthen outbreak preparedness and ensure equitable access to resources to build resilience against emerging infectious diseases.

## Graphical abstract

- The WHO declares the Mpox outbreak a Public Health Emergency of International Concern, with the DRC as the epicenter in the EAC.
- DRC, Uganda, and Burundi experience extensive transmission; Kenya and Uganda show higher case fatality rates.
- Containment measures include surveillance, public awareness, infection prevention training, and vaccination (>73% of 801,000 doses in DRC).
- Limited healthcare infrastructure and misinformation hinder control, highlighting the need for a coordinated regional strategy and equitable resource access.

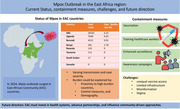

## 1 Introduction

Mpox, formerly known as monkeypox, is a zoonotic disease caused by the monkeypox virus (MPXV), which emerged as a global health threat due to the high transmission and challenges to the health systems [1, 2]. Mpox is a double‐stranded DNA virus that belongs to the Poxviridae family of the genus *Orthopoxvirus* and has two different clades, Clade I historically associated with the Congo Basin and Clade II associated with West Africa outbreaks [3]. Mpox virus was initially identified in captive monkeys in Denmark in 1959, and the first human case was identified in 1970 from a male in the Democratic Republic of the Congo (DRC) [4]. By 1980, 59 cases were reported in DRC [5], and in the following decades, Mpox sporadic outbreaks heavily affected Central and West Africa, especially in DRC, Nigeria, Cameroon, and the Central African Republic [6]. In 2017–2018, Nigeria recorded the largest number of cases, over 276 confirmed cases, with human‐to‐human transmission [7]. In 2022, Mpox outbreak marked a significant shift in the disease's epidemiology. It showed the change from sporadic cases, primarily linked to animal contact, to widespread human‐to‐human transmission [8, 9].

On August 13, 2024, the Africa Centre for Disease Control and Prevention declared Mpox as a Public Health Emergency of Continental Security (PHECS) [10, 11]. The following day, the World Health Organization (WHO) declared Mpox a Public Health Emergency of International Concern (PHEIC) in response to the high number of cases in DRC and the ongoing regional spread [12, 13, 14]. This declaration highlighted the urgent need for increased surveillance, vaccination efforts, and international collaboration to contain the outbreak [15].

The epicenter of the 2024 Mpox outbreak was the DRC, which is a member of the East African Community (EAC), accounting for 96% of new cases and deaths [16]. The EAC is currently a global Mpox hotspot as partner states were directly affected by Mpox outbreak [17]. The EAC is composed of eight countries, namely, (i) the Republic of Burundi, (ii) the DRC, (iii) the Republic of Kenya, (iv) the Republic of Rwanda, (v) the Federal Republic of Somalia, (vi) the Republic of South Sudan, (vii) the Republic of Uganda, and (viii) the United Republic of Tanzania [18]. Mpox outbreak in the DRC began in April 2024 and rapidly spread to neighboring countries (Burundi, Uganda, Kenya, and Rwanda), which had not previously experienced Mpox. In addition, a travel‐associated clade Ib case was reported in Sweden in August 2024 [11]. Several countries beyond the EAC region have confirmed Mpox cases linked to EAC countries [19]. In Southern Africa, Zambia and Zimbabwe reported travel‐related cases [20]. In Europe, a travel‐associated Clade Ib case was reported in Sweden in August 2024 [11]. Moreover, Germany reported seven cases (three linked to EAC), whereas the United Kingdom and Northern Ireland both confirmed six cases, all linked to EAC. In Asia, China recorded seven cases with one linked to DRC, and Thailand confirmed one case linked to EAC. In North America, the USA confirmed four cases and Canada one case associated with EAC countries [19]. These cases around the world show the importance of coordinated global surveillance and response efforts.

Most countries in the EAC have been reporting Mpox cases, with the DRC, Burundi, and Uganda reporting the highest numbers. In response, the EAC implemented strategies to control Mpox, focusing on monitoring cross‐border movements and addressing health challenges arising from integrated public health frameworks [17, 21]. However, systemic gaps in healthcare infrastructure, different levels of disease surveillance capacity, and sociopolitical instability in some EAC member states complicated the region's response [11, 22, 23]. Furthermore, the stigma associated with Mpox, misinformation, and limited vaccine accessibility presented further barriers to effective containment [24]. Addressing these challenges requires a comprehensive approach that includes enhancing healthcare infrastructure, improving public awareness to prevent stigma and misinformation, and ensuring equitable access to vaccines across all member states. Therefore, this review aimed to provide a comprehensive analysis of Mpox outbreak in the EAC region, which has been ongoing since 2024, by focusing on epidemiological trends, containment measures, and challenges. By reflecting on the successes and challenges experienced, this review seeks to provide actionable insights for strengthening future outbreak preparedness and adopting regional resilience to emerging infectious diseases.

## 2 Status and Containment Measures of Mpox in the EAC Member States

Mpox continues to affect most EAC countries, with considerable variations in transmission patterns, case severity, and the populations at risk (Table 1). As of July 2025, DRC remains the most affected country, contributing the largest proportion of regional Mpox cases (28,165 cases and 69 deaths), with widespread transmission across nearly all provinces [25, 26]. Uganda and Burundi also experienced sustained community transmission, whereas Kenya had a disproportionately high case fatality rate (CFR) when compared to reported cases. Rwanda, Tanzania, and South Sudan documented relatively low numbers of confirmed cases, with no Mpox‐related deaths. Somalia remains the only EAC country that has not reported any Mpox case, possibly due to limited laboratory testing/surveillance system or limited introduction of the virus from neighboring countries. Differences of Mpox burden in EAC countries could be explained by multiple factors, including proximity to high‐burden countries, the level of implementation of testing and control measures, and the demographic characteristics of exposed populations.

**TABLE 1:** Epidemiological status of Mpox in East African Community (EAC).

S/N	Country	Total cases	Total deaths	CFR (%)	Most affected age group
1.	DRC	28,165	69	0.25	Multiple age groups
2.	Uganda	7648	48	0.63	20–29 years
3.	Burundi	4231	1	0.02	Children ≤15 (39.5%)
4.	Kenya	281	5	1.78	Multiple age groups
5.	Rwanda	124	0	0	Multiple age groups
6.	Tanzania	111	0	0	Multiple age groups
7.	South Sudan	17	0	0	Multiple age groups
8.	Somalia	0	0	0	Multiple age groups

Abbreviations: CFR, case fatality rate; DRC, Democratic Republic of Congo.

### 2.1 Status of Mpox in the DRC

Since April 2024, the DRC has experienced a significant Mpox outbreak, characterized by continual human‐to‐human transmission and the circulation of multiple viral subclades in the community [27]. The outbreak started with Mpox Clade Ib in South Kivu province which was rapidly transmitted to North Kivu and nearly all regions of the country, affecting 22 of 26 provinces [28, 29]. Mpox outbreak was complicated by co‐circulation of both subclades Ia and Ib, along with multiple introductions in densely populated areas [30]. Moreover, genetic alteration of Clade Ib increased transmissibility, mostly linked to sexual transmission networks and significant spread within the community [31].

The most affected provinces are Equateur, North Kivu, Sankuru, South Kivu, South‐Ubangi, and Tshopo [29]. The main risk factors included sexual networks involving men who have sex with men (MSM) and sex workers [32, 33, 34]. However, nonsexual exposures, such as hunting, fishing, agriculture, wildlife trade, poverty, and contact with an infected individual, have been previously cited [35, 36].

Containment measures, including vaccination campaigns, contact tracing, enhanced surveillance, case isolation, and infection prevention and control (IPC) strategies, were adopted [37]. Indeed, the Modified Vaccinia Ankara‐Bavarian Nordic (MVA‐BN) vaccine has been deployed, and a vaccination model proved that immunizing 80% of <15 children in the DRC could reduce illness, viral circulation, and mortality [38]. Overall vaccine acceptance in the DRC was 61.0%, with higher willingness reported among healthcare workers and individuals from endemic regions, underscoring the need to boost community vaccination awareness campaigns [39]. The country has administered over 73% of all Mpox vaccines delivered in Africa [40]. The screening capacity was constrained by shortage of medical supplies and limited staffing, resulting in increased turnaround time (TAT) [41]. The TAT was later reduced from 10 days to 24–48 h [41, 42] due to improved supply of sample collection kits, personal protective equipment, and decentralization of laboratory capacity.

### 2.2 Status of Mpox in Burundi

Burundi experienced a rapid geographical spread of Clade Ib MPXV [40] reaching nearly all health districts by early 2025, with the highest transmission in Bujumbura and Gitega [43, 44]. The Clade Ib MPXV reported in Burundi had been circulating in South Kivu [45], suggesting cross‐border movement of people in the region. Although the national CFR remained low, Mpox was more prevalent among children under 15, highlighting distinct demographic vulnerability [28].

Burundi's response initiatives included a large‐scale IPC training, providing psychosocial support to affected individuals, expansion of WASH services in schools, establishment of isolation centers, and community sensitization [46]. The government, in collaboration with Africa CDC, initiated plans to introduce vaccination targeting high‐risk groups, such as health workers, sex workers, MSM, eco‐guards/hunters, and other high‐risk groups [47]. Such measures enabled Burundi to control Mpox through a strong system resulting in a low CFR.

### 2.3 Mpox Status in Uganda

Uganda reported geographical spread, particularly with high transmission in Kampala and Wakiso [36]. After a few days of confirming the first two cases, the outbreak was declared with 41 confirmed cases, revealing a high transmission rate in Uganda [48, 49]. The outbreak was exclusively driven by Clade Ib MPXV, mostly affecting young adults (18–29 years) and males. The outbreak continued to spread rapidly and widely, affecting 82% of the districts, with a constant resurgence in high‐burden districts [50, 51]. Similar to other areas affected by Clade Ib, immunocompromised individuals were greatly affected, and 47.9% of Mpox‐related deaths occurred in HIV‐coinfected people [50].

The Ugandan Ministry of Health implemented extensive containment measures, including early detection, contact tracing to promptly identify and isolate cases, public health awareness campaigns, and the establishment of treatment units in affected districts [52, 53]. Early in the outbreak, the introduction of Mpox cases into schools led to joint efforts of the Ministry of Health and the Ministry of Education to enhance contact tracing and develop disease control protocols specific to schools [48, 49]. Within communities, outbreak response was reinforced by training field responders, building capacity of laboratories, and enforcing electronic surveillance systems, all of which promoted early detection and treatment [52]. Vaccination efforts began in the capital city of Uganda (Kampala), prioritizing high‐risk populations and healthcare workers, and it was supported by vaccine donations from Africa CDC and the European Commission [54].

### 2.4 Status of Mpox in Rwanda

In Rwanda, the outbreak began in July 2024, with early cases linked to travel history from the DRC before the onset of symptoms [46]. Transmission remained comparatively limited, with no recorded deaths. Clade Ib MPXV was identified as the circulating strain in Rwanda, consistent with regional trends [50, 55]. Rwanda, through the Rwanda Biomedical Centre (RBC), put efforts in advanced diagnostic capacity by implementing genomic sequencing and bioinformatics analysis as key measures in surveillance of infectious and outbreak diseases [24].

Rwanda's response emphasizes strong diagnostic capacity, including polymerase chain reaction (PCR) testing for all suspected cases, genomic sequencing, and decentralized testing hubs. Confirmation of MPXV infection was based on nucleic acid amplification testing (NAAT), and positive samples were sequenced [56]. Three testing hubs were established in different provinces to promote proximity of testing services to the high‐risk sites. Additionally, surveillance was strongly enhanced through contact tracing and regular follow‐up of high‐risk individuals [50, 55]. A few months after the outbreak declaration, Rwanda launched a vaccination campaign against Mpox in September 2024, targeting health workers, cross‐border business operators, hospitality workers, truck drivers, and sex workers [57].

### 2.5 Mpox Status in Kenya

Kenya reported Mpox outbreak in July 2024, with cases concentrated in coastal and border counties, such as Nakuru, Mombasa, and Busia [58, 59]. Most of the confirmed cases were reported from truck drivers or their close contacts, and 63% of all cases were mostly linked to sexual transmission [60, 61]. Sequencing of Clade Ib Mpox isolated from the index Kenyan case, who was a truck driver with travel history to Uganda, revealed that the virus was linked to the regional outbreak [60, 61]. Although overall caseload remains lower than in neighboring countries, the CFR is relatively high in Kenya, suggesting potential delays in seeking care or severity among affected populations. Clade Ib is the circulating strain in Kenya, and only human‐to‐human transmission has been recorded [60, 61].

Response activities included public awareness campaigns to educate communities on Mpox prevention, WASH interventions, and multisectoral resource mobilization. Most of the containment measures were directed to high‐risk counties, where case detection was enhanced through deployment of rapid response teams and large‐scale travelers screening at entry points [62]. However, response was initially hindered by low public risk perception and inadequate resources, which were mitigated through community awareness campaigns and supplies for infection prevention in congregated settings [63]. Nonetheless, the number of cases are steadily increasing, undermining the effectiveness of control measures, probably due to the high cross‐border mobility and failure to identify all high‐risk individuals. All Mpox‐related deaths in Kenya were recorded among HIV‐coinfected individuals, highlighting that PLHIV constitute a vulnerable group needing much care to contain the outbreak.

### 2.6 Status of Mpox in the United Republic of Tanzania

Tanzania confirmed its first Mpox cases in March 2025, involving individuals in Dar es Salaam [36, 64, 65, 66]. Similar to Kenya, Mpox was introduced by a truck driver, leading to border screening and case investigation. However, community transmission persisted [25, 64, 65]. The dominance of Clade Ib Mpox, coupled with its high transmissibility, contributed to the widespread of the virus in different countries; however, no deaths have been reported so far [25].

Tanzania adopted various containment measures including strengthened port health services, border screening, community surveillance, health education campaigns, and prioritization of vaccination for vulnerable populations such as PLHIV [67, 68]. The country also benefited from the EAC initiative of deploying mobile laboratories for strengthening surveillance at the borders to prevent the spread of infection and conducting genomic surveillance among the EAC members [17].

### 2.7 Status of Mpox in South Sudan

South Sudan confirmed its first Mpox case in February 2025, mostly travel‐related, with no deaths to date [25, 36]. This person was a Ugandan national living in Juba and was confirmed by the National Public Health Laboratory (NPHL) [69]. Clade Ib is the circulating strain, consistent with reports in other countries [69].

The Ministry of Health coordinated responses, including contact tracing, strengthened surveillance at major entry points, laboratory capacity building, and the provision of IPC and WASH resources at the community and facility levels [17, 70].

### 2.8 Status of Mpox in Somalia

Somalia has not yet reported any confirmed Mpox cases as of November 2025 [71]. This may be linked to a weakened health system and limited testing capacity following more than 25 years of civil war and instability. However, the country remains vigilant at its borders and is strengthening its health infrastructure, to migitage the risk of Mpox virus spread from neighboring regions [71]. Somalia, like other countries in EAC, where healthcare infrastructure and disease surveillance are not advanced, requires greater efforts to address and prepare for outbreaks like Mpox [72, 73].

The country is strengthening preparedness through training of healthcare workers, laboratory scientists, and epidemiologists who will help in the implementation of managing outbreaks in Somalia [71]. Additionally, the Ministry of Health launched an awareness campaign to educate the public about Mpox symptoms, prevention, and transmission.

Table 2 summarizes Mpox containment measures in the EAC that include enhanced surveillance, contact tracing, public awareness campaigns, and vaccination efforts. Mobile laboratories support high‐risk areas, and regional collaboration strengthens cross‐border surveillance. Challenges such as limited vaccine access and misinformation remain significant barriers.

**TABLE 2:** Containment measures and challenges while responding to Mpox outbreak.

Country	Key containment measures	Mpox vaccine started	Challenges
Rwanda	Surveillance, contact tracing, public health campaigns, vaccination rollout, and establishment of a testing hub	Yes	Vaccine shortages
Burundi	Training healthcare workers, psychosocial support, IPC, and WASH interventions	No	High transmission rate
Uganda	Early detection, contact tracing, treatment units, public awareness campaigns	Yes	High CFR
Kenya	Public awareness campaigns, WASH initiatives, and resource mobilization	No	Transmission along trade routes
Tanzania	Port health services, community surveillance, public health education	No	Potential undetected cases
DRC	Vaccination campaigns, contact tracing, IPC strengthening, and enhanced surveillance	Yes	Widespread cases
South Sudan	Contact tracing, training of laboratory staff, strengthened surveillance at entry points, IPC, and WASH reinforcement	No	Limited laboratory capacity, fragile health system
Somalia	Health‐worker training, public awareness campaigns, border vigilance, and surveillance	No	Weak surveillance infrastructure, risk of undetected introduction

Abbreviations: CFR, case fatality rate; DRC, Democratic Republic of Congo; IPC, infection prevention and control.

## 3 Comparative Analysis of Epidemiological Differences Across EAC Countries

Epidemiological outcomes across EAC member states show marked variation, driven by differences in surveillance systems, demographic profiles, healthcare access, and local transmission dynamics. Apart from DRC, which had an ongoing of Mpox outbreak, Burundi, Kenya, Rwanda, and Uganda all reported their first cases in 2024. Contrarily, Tanzania and South Sudan have limited documentation on how the Mpox outbreak was managed.

In addition, apart from DRC known to be the epicenter of the Mpox outbreak, Kenya reported a higher CFR (1.78%) compared to 0.02% for Burundi, which may be influenced by several factors. Kenya's affected population includes more adults and mobile populations along major trade routes [60, 61], which may contribute to delayed care‐seeking or higher prevalence of comorbidities. In contrast, Burundi reported that Mpox was predominant in a younger population (children under 15) who generally presented with milder disease. So far, no deaths have been reported in Rwanda, Tanzania, and South Sudan. It is important to mention that CFR estimates depend strongly on case detection and death reporting systems, which vary widely among EAC countries.

Differences in surveillance and diagnostic capacity also contribute to heterogeneous outcomes [24]. For example, Rwanda and Uganda, through the EAC, benefited from strengthened laboratory and surveillance networks, including genomic sequencing and decentralized testing hubs [17]. Furthermore, fragile health systems and conflict‐related disruptions in the Eastern Part of the DRC, South Sudan, and Somalia may limit access to timely care and complicate diagnosis. Lastly, the degree of urbanization and mobility patterns, including high‐traffic transport corridors between Kenya, Uganda, and Tanzania, shape exposure risk and influence the outbreak severity. These variations highlight the need for tailored, country‐specific response strategies while reinforcing regional coordination across the EAC.

## 4 Challenges and Future Directions

Despite ongoing response efforts, several challenges continue to hinder effective Mpox control in East Africa and other regions. One major barrier is unequal vaccine access, which has led to increased cases and deaths in countries with limited supply or delayed vaccination rollouts [74]. This inequity reflects broader systemic gaps in global distribution of health resource [75]. Vaccine disparities represent not only logistical failures but also ethical shortcomings in justice and global solidarity, where populations in low‐resource settings are consistently last to receive life‐saving interventions [76].

Misinformation regarding Mpox is also increasing, especially on social media platforms. On X (formerly Twitter), large volumes of misleading or false information circulate about the transmission of the virus, its severity, and origins [77, 78, 79]. Such misinformation not only undermines public awareness but also weakens the trust in different health interventions and institutional strategies to combat Mpox in different countries. This critical challenge needs much attention to ensure effective disease control in the community [24]. Transparent communication and trust‐building are essential pillars of outbreak response, and failure to address misinformation threatens both public accountability and community resilience [76].

Mpox‐related stigma remains another major challenge globally. Stigma contributes to delayed healthcare seeking, nondisclosure of the disease, discrimination in some health settings, and affects mental health [80, 81]. In Burundi, a motorcyclist recovering from Mpox experienced severe stigma resulting in social isolation, loss of livelihood, and housing instability due to visible scars [82]. Political instability affects the mitigation strategies to control Mpox in the region [83]. In settings such as the DRC, South Sudan, and Somalia, insecurity disrupts humanitarian operations, limits access to healthcare, and complicates disease surveillance, laboratory diagnostics, and treatment [11].

Infrastructure and laboratory capacity remain critical gaps across many EAC countries. Limited availability of genomic sequencing platforms, testing kits, and bioinformatics expertise delays identification of circulating clades in the region and reduces the implementation of public health interventions [11]. Although Africa CDC, WHO, and other partners pledged to increase vaccine availability across the continent, logistic challenges including cold chain requirements and transport constraints, continue to impede equitable distribution [84].

To strengthen future Mpox preparedness and response, EAC member states must combine operational improvements with an ethically grounded framework that emphasizes justice, solidarity, and respect for rights. By improving vaccine equity, combating misinformation through transparent communication, addressing stigma, expanding laboratory capacity, and enhancing cross‐border collaboration, the region can significantly improve its resilience and ensure a more equitable and effective response to emerging infectious diseases.

## 5 Conclusion

The Mpox outbreak surged in 2024 and remains ongoing in the EAC region, with the DRC and Burundi being the most affected countries. Major containment measures were implemented, including monitoring cross‐border movement and improving testing capacity and vaccination coverage. This outbreak serves as another wake‐up call for the EAC to strengthen public health responses to recurrent emerging infectious diseases. Although significant progress was made in containing the outbreak, addressing systemic gaps in healthcare infrastructure and resource distribution remains essential. The EAC must prioritize investments in health systems, advance international partnerships, and influence community‐driven approaches to build resilience and ensure equitable health outcomes across the region.

## Author Contributions

Reuben Sindayiheba and Noel Gahamanyi contributed to the design of the work, data collection, analysis, and drafting of the manuscript. Edouard Ntagwabira, Emmanuel Kabalisa, Gashegu Misbah, Isabelle Mukagatare, Leon Mutesa, and Claude Mambo Muvunyi significantly revised the manuscript and supervised the work. All authors read and approved the final version of the manuscript.

## Funding

The authors have nothing to report.

## Disclosure

The authors have nothing to report.

## Ethics Statement

The authors have nothing to report.

## Consent

The authors have nothing to report.

## Conflicts of Interest

The authors declare no conflicts of interest.

## Data Availability

Data sharing not applicable to this article as no datasets were generated or analyzed during the current study.

## References

[1] O. Mitjà , A. Alemany , M. Marks , et al., “Mpox in People With Advanced HIV Infection: A Global Case Series,” Lancet 401, no. 10380 (2023): 939–949, 10.1016/S0140-6736(23)00273-8. 36828001

[2] D. B. Olawade , O. Z. Wada , S. C. Fidelis , et al., “Strengthening Africa's Response to Mpox (Monkeypox): Insights From Historical Outbreaks and the Present Global Spread,” Science in One Health 3 (2024): 100085, 10.1016/j.soh.2024.100085. 39583938 PMC11582772

[3] A. Karagoz , H. Tombuloglu , M. Alsaeed , et al., “Monkeypox (Mpox) Virus: Classification, Origin, Transmission, Genome Organization, Antiviral Drugs, and Molecular Diagnosis,” Journal of Infection and Public Health 16 (2023): 531–541, 10.1016/j.jiph.2023.02.003. 36801633 PMC9908738

[4] C. L. J. Ugwu , N. L. Bragazzi , J. Wu , et al., “Risk Factors Associated With Human Mpox Infection: A Systematic Review and Meta‐Analysis,” BMJ Global Health 10, no. 2 (2025): e016937, 10.1136/bmjgh-2024-016937. PMC11795413 39900427

[5] S. Hasan and S. Saeed , “Monkeypox Disease: An Emerging Public Health Concern in the Shadow of COVID‐19 Pandemic: An Update,” Tropical Medicine and Infectious Disease 7 (2022): 283, 10.3390/tropicalmed7100283. 36288024 PMC9607171

[6] E. M. Bunge , B. Hoet , L. Chen , et al., “The Changing Epidemiology of Human Monkeypox—A Potential Threat? A Systematic Review,” PLOS Neglected Tropical Diseases 16, no. 2 (2022): e0010141, 10.1371/journal.pntd.0010141. 35148313 PMC8870502

[7] A. Yinka‐Ogunleye , O. Aruna , M. Dalhat , et al., “Outbreak of Human Monkeypox in Nigeria in 2017–18: A Clinical and Epidemiological Report,” Lancet Infectious Diseases 19, no. 8 (2019): 872–879, 10.1016/S1473-3099(19)30294-4. 31285143 PMC9628943

[8] WHO , Multi‐Country Monkeypox Outbreak in Non‐Endemic Countries (WHO, 2022).

[9] F. M. Lum , A. Torres‐Ruesta , M. Z. Tay , et al., “Monkeypox: Disease Epidemiology, Host Immunity and Clinical Interventions,” Nature Reviews Immunology 22, no. 10 (2022): 597–613, 10.1038/s41577-022-00775-4. PMC9443635 36064780

[10] Africa CDC , Africa CDC Declares Mpox A Public Health Emergency of Continental Security, Mobilizing Resources Across the Continent (Africa CDC, 2024).

[11] F. O. Rahim , M. Fallah , U. Jain , et al., “Challenges and Ongoing Actions to Address the Mpox Emergency in Africa,” Annals of Global Health 90 (2024): 68, 10.5334/aogh.4580. 39554695 PMC11568800

[12] WHO , WHO Director‐General Declares Mpox Outbreak a Public Health Emergency of International Concern (WHO, 2024). PMC11376700 39218470

[13] P. Adepoju , “Mpox Declared a Public Health Emergency,” Lancet (London, England) 404, no. 10454 (2024): E1–E2. 39182498 10.1016/S0140-6736(24)01751-3

[14] N. Ndembi , M. O. Folayan , N. Ngongo , et al., “Mpox Outbreaks in Africa Constitute a Public Health Emergency of Continental Security,” Lancet Global Health 12, no. 10 (2024): e1577–e1579, 10.1016/S2214-109X(24)00363-2. 39178878

[15] N. Kaur , J. Dabar , and P. Bassi , “Monkeypox: A Re‐Emerging Disease,” Indian Journal of Pharmacology 56 (2024): 129–135, 10.4103/ijp.ijp_156_23. 38687317 PMC11161004

[16] Africa CDC , Mpox Outbreaks in Africa Constitute a Public Health Emergency of Continental Security (Africa CDC, 2024). 10.1016/S2214-109X(24)00363-2 39178878

[17] F. Gehre , E. Nzeyimana , H. I. Lagu , et al., “Rapid Regional Mobile Laboratory Response and Genomic Monkeypox Virus (MPXV) Surveillance in Seven East African Community Partner States, August 2024: Preparedness Activities for the Ongoing Outbreak,” Eurosurveillance 29, no. 35 (2024): 2400541, 10.2807/1560-7917.ES.2024.29.35.2400541. 39212058 PMC11484336

[18] F. Gehre , H. I. Lagu , E. Achol , et al., “The East African Community Mobile Laboratory Network Prepares for Monkeypox Outbreaks,” Journal of Public Health in Africa 14, no. 6 (2023): 2309, 10.4081/jphia.2023.2309. 37680705 PMC10481785

[19] WHO , Mpox: Multi‐Country External Situation Report #48 ‐ 10 March 2025 (WHO 2025).

[20] WHO , Mpox: Multi‐Country External Situation Report #45 ‐ 11 January 2025 (WHO, 2025).

[21] Africa CDC , Mpox Surveillance Reporting Protocol for African Union Member States (Africa CDC, 2024).

[22] G. P. Gómez‐Pérez , S. O. O. Sam , N. Ndembi , and T. F. R. De Wit , “Mpox After COVID‐19 in Africa: Different Epidemic, Similar Challenges,” Journal of Public Health in Africa 16, no. 1 (2025): 874. 40182747 10.4102/jphia.v16i1.874 PMC11966709

[23] N. A. Evaborhene , J. O. Oga , Y. A. Adebayo , et al., “Caught Between Violence: Mpox Virus and the Perils of Neglect in Africa,” BMJ Global Health 9, no. 11 (2024): 1–5, 10.1136/bmjgh-2024-017090. PMC11574508 39557449

[24] P. Gashema , T. Musafiri , F. Ndahimana , et al., “Mpox in East Africa: Learning From COVID‐19 and Ebola to Strengthen Public Health Responses,” Viruses 16 (2024): 1578, 10.3390/v16101578. 39459912 PMC11512314

[25] WHO , Mpox: Multi‐Country External Situation Report No.52 (WHO, 2025).

[26] WHO , Multi‐Country‐Outbreak‐of‐Mpox–External‐Situation‐Report–56 (WHO, 2025), https://www.who.int/publications/m/item/multi‐country‐outbreak‐of‐mpox–external‐situation‐report–56‐31‐july‐2025.

[27] D. V. Parums , “Editorial: Reasons for Increasing Global Concerns for the Spread of Mpox,” Medical Science Monitor: International Medical Journal of Experimental and Clinical Research 30 (2024): e946343. 39217431 10.12659/MSM.946343 PMC11376131

[28] A. Nizigiyimana , F. Ndikumwenayo , S. Houben , et al., “Epidemiological Analysis of Confirmed Mpox Cases, Burundi, 3 July to 9 September 2024,” Euro Surveillance 29, no. 42 (2024): 2400647, 10.2807/1560-7917.ES.2024.29.42.2400647. 39421954 PMC11487916

[29] I. Salomon , A. E. Hamitoglu , U. Hertier , et al., “Monkeypox Outbreak in the Democratic Republic of Congo: A Comprehensive Review of Clinical Outcomes, Public Health Implications, and Security Measures,” Immunity, Inflammation and Disease 12 (2024): e70102, 10.1002/iid3.70102. 39679843 PMC11647977

[30] T. Wawina‐Bokalanga , P. Akil‐Bandali , E. Kinganda‐Lusamaki , et al., “Co‐Circulation of Monkeypox Virus Subclades Ia and Ib in Kinshasa Province, Democratic Republic of the Congo, July to August 2024,” Eurosurveillance 29, no. 38 (2024): 2400592, 10.2807/1560-7917.ES.2024.29.38.2400592. 39301745 PMC11484285

[31] S. Srivastava , Laxmi , K. Sharma , et al., “Clade Ib: A New Emerging Threat in the Mpox Outbreak,” Frontiers in Pharmacology 15 (2024): 1504154, 10.3389/fphar.2024.1504154. 39749207 PMC11693458

[32] T. Hrynick and M. Schmidt‐Sane , Roundtable Report: Discussion on Mpox in DRC and Social Science Considerations for Operational Response (Social Science in Humanitarian Action Platform (SSHAP), 2024).

[33] E. M. Kibungu , E. H. Vakaniaki , E. Kinganda‐Lusamaki , et al., “Clade I–Associated Mpox Cases Associated With Sexual Contact, the Democratic Republic of the Congo,” Emerging Infectious Diseases 30, no. 1 (2024): 172–176, 10.3201/eid3001.231164. 38019211 PMC10756366

[34] L. M. Masirika , J. C. Udahemuka , L. Schuele , et al., “Mapping and Sequencing of Cases From an Ongoing Outbreak of Clade Ib Monkeypox Virus in South Kivu, Eastern Democratic Republic of the Congo Between September 2023 to June 2024.” preprint, medRxiv, September 19, 2024.

[35] K. Y. Dieu‐Merci , B. Singa Valentin , B. L. Ley , et al., “Mpox: Epidemiological Profile and Factors Associated With Its Emergence in the Isangi Territory, Democratic Republic of Congo,” International Journal of Applied Science and Engineering Review 05, no. 05 (2024): 27–43, 10.52267/IJASER.2024.5503.

[36] World Health Organization , Global Mpox Trends (WHO, 2025).

[37] M. R. Bashwira , I. M. Mihigo , and D. Duclos , Key Considerations: Mpox, Mining, and Vulnerabilities of Women and Children in Eastern DRC (SSHAP, 2024).

[38] A. Savinkina , J. Kindrachuk , I. I. Bogoch , et al., “Modelling Vaccination Approaches for Mpox Containment and Mitigation in the Democratic Republic of the Congo,” Lancet Global Health 12, no. 12 (2024): e1936–e1944, 10.1016/S2214-109X(24)00384-X. 39393385

[39] S. Petrichko , J. Kindrachuk , D. Nkamba , et al., “Mpox Vaccine Acceptance, Democratic Republic of the Congo,” Emerging Infectious Diseases 30, no. 12 (2024 1): 2614–2619, 10.3201/eid3012.241226. 39486156 PMC11616662

[40] WHO , Mpox Epidemiological Update (WHO, 2025).

[41] WHO Reginal Office for Africa , Improving Mpox Diagnosis in DRC Amidst the Outbreak (WHO Reginal Office for Africa, 2024).

[42] UNICEF , Democratic Republic of Congo Humanitarian Situation Report No. 7: Mpox Response‐31 March 2025 (UNICEF, 2025).

[43] L. Murhula Masirika , J. Claude Udahemuka , L. Schuele , et al., “Ongoing Mpox Outbreak in Kamituga, South Kivu Province, Associated With Monkeypox Virus of a Novel Clade I Sub‐Lineage, Democratic Republic of the Congo, 2024,” Eurosurveillance 29, no. 11 (2024): 2400106, 10.2807/1560-7917.ES.2024.29.11.2400106. 38487886 PMC10941309

[44] UNICEF , Burundi Humanitarian Situation Report No. 3 (Mpox Level 3 Emergency), 31 October 2024 (UNICEF, 2024).

[45] I. Brosius , E. H. Vakaniaki , G. Mukari , et al., “Epidemiological and Clinical Features of Mpox During the Clade Ib Outbreak in South Kivu, Democratic Republic of the Congo: A Prospective Cohort Study,” Lancet 405, no. 10478 (2025): 547–559, 10.1016/S0140-6736(25)00047-9. 39892407 PMC7618259

[46] UNICEF , Burundi Humanitarian Situation Report No. 4 (Mpox Level‐3 Emergency) (1–30 November 2024) (UNICEF, 2024).

[47] Africa CDC , Burundi Revises Outbreak Response Plans (Africa CDC, 2025).

[48] UNICEF , Uganda Mpox Level 3 Emergency—Situation Report, 30 September 2024 (UNICEF, 2024).

[49] D. Wenani , A. Kamukama , E. Namulondo , et al., “Epidemiological Characteristics and Transmission Dynamics of the First 66 Confirmed Mpox Cases, Nakasongola District, Uganda, September−November 2024,” Uganda Public Health Bulletin 10, no. 1 (2025): 1.

[50] I. Abubakar , J. Lutwama , C. Kyobutungi , and O. Sankoh , “Mpox Global Emergency: Strengthening African Leadership,” Lancet 404 (2024): 1286–1288, 10.1016/S0140-6736(24)02068-3. 39312930

[51] MOH of Uganda , National Mpox Situation Report –31 July 2025 (MOH of Uganda, 2025).

[52] WHO Africa , Reinforcing Outbreak Response Systems to Curb Mpox in Uganda (WHO Africa, 2024).

[53] WHO Africa , Mpox Outbreak in Uganda Situation Update –24 October 2024 (WHO Africa, 2024).

[54] Xinhua , Uganda Starts Mpox Vaccination Targeting High‐Risk Populations (Xinhua, 2025).

[55] WHO , Mpox—African Region (WHO, 2024).

[56] RBC , Rwanda National Guideline for Surveillance and Management of Mpox (RBC, 2024).

[57] L. Schnirring , Rwanda Launches Mpox Vaccine Campaign (CIDRAP, 2024).

[58] WHO Africa , Kenya Conducts a Multisectoral Intra‐Action Review to Improve Response to Ongoing Mpox Outbreak (WHO Africa, 2024), https://www.afro.who.int/countries/kenya/news/kenya‐conducts‐mul.

[59] N. Ndembi , M. O. Folayan , A. Komakech , et al., “Evolving Epidemiology of Mpox in Africa in 2024,” New England Journal of Medicine 392, no. 7 (2025): 666–676, 10.1056/NEJMoa2411368. 39887004

[60] P. Mutuku , A. Abade , M. Owiny , et al., “Clade Ib Mpox Outbreak—Kenya, July 2024–February 2025,” MMWR Morbidity and Mortality Weekly Report 74, no. 22 (2025): 379–384, 10.15585/mmwr.mm7422a2. 40531681 PMC12176102

[61] S. K. Langat , K. Gathii , K. Limbaso , et al., “Complete Genome of an Mpox Clade 1b Virus From Kenya,” Microbiology Resource Announcements 14, no. 5 (2025): 8–11, 10.1128/mra.00050-25. PMC12060682 40214247

[62] WHO Regional Office for Africa , Kenya Steps Up Surveillance, Response Measures to Curb Mpox (WHO Regional Office for Africa, 2024).

[63] UNICEF Kenya , Kenya Humanitarian Situation Report No. 2: Mpox Level 3 Emergency, 31 October 2024 (UNICEF Kenya, 2024).

[64] Ministry of Health (United Republic of Tanzania) , Taarifa kwa Umma Kuhusu Ugonjwa wa Mpox Nchini (Ministry of Health (United Republic of Tanzania), 2025).

[65] WHO Africa , United Republic of Tanzania Declares First Cases of Mpox (WHO Africa, 2025).

[66] J. J. Mhagama and Jamhuri ya Muungano wa Tanzania Wizara ya Afya , Taarifa Kwa Umma Kuhusu Ugonjwa Wa Mpox Nchini (Jamhuri ya Muungano wa Tanzania Wizara ya Afya, 2025).

[67] Ministry of Health (United Republic of Tanzania) , Presss Release of Mpox Threat (Ministry of Health (United Republic of Tanzania), 2024).

[68] M. M. Mshenga , I. A. Mussa , and S. H. Haji , “Public Health Response to Mpox: Safeguarding Vulnerable Key Populations and People Living With HIV in Zanzibar,” AIDS Research and Therapy 21 (2024): 65, 10.1186/s12981-024-00658-9. 39343958 PMC11440885

[69] B. Takpiny , South Sudan Confirms 1st Case of Mpox (Anadolu Ajansı, 2025).

[70] Y. A. D. Juach , National Monkeypox Preparedness and Response Plan From July 2024 to June 2026 (IOM South Sudan, 2024).

[71] F. S. Mohamed , O. J. Okesanya , M. M. Ahmed , A. A. Hirey , B. Garba , and A. S. Ali , “Enhancing Epidemic Preparedness in Somalia: Lessons From Mpox Outbreaks in East Africa,” Journal of Taibah University for Science 19, no. 6 (2024): 1130–1131. 10.1016/j.jtumed.2024.12.004 PMC11719359 39802217

[72] M. M. Ahmed , N. I. Dirie , O. J. Okesanya , and D. E. Lucero‐Prisno , “Mpox in East Africa: Confronting a Public Health Emergency,” Journal of Taibah University Medical Sciences 19 (2024): 992–994, 10.1016/j.jtumed.2024.09.010. 39435013 PMC11491704

[73] A. Yusuf Ali , A. A. Mohamed , O. O. Eyibio , and N. S. George , “Health Under Siege: Mpox and the Populations on the Ethiopia–Somalia–Kenya Border,” Public Health Challenges 4 (2025): e70061, 10.1002/puh2.70061.

[74] A. Zumla , P. J. Rosenthal , N. A. Sam‐Agudu , et al., “The 2024 Public Health Emergency of International Concern: A Global Failure to Control Mpox,” American Journal of Tropical Medicine and Hygiene 112, no. 1 (2025): 17–20, 10.4269/ajtmh.24-0606. PMC11720770 39406210

[75] A. N. Cynthia and G. T. Nchanji , “MPOX Outbreak in Africa: The Urgent Need for Local Manufacturing of the Vaccine and Decolonized Health Systems,” BMC Public Health [Electronic Resource] 25, no. 1 (2025): 3838, 10.1186/s12889-025-25120-x. 41204158 PMC12595634

[76] A. E. Obasa , M. Botes , A. Kleinsmidt , and C. Staunton , “Mpox and the Ethics of Outbreak Management: Lessons for Future Public Health Crises,” Developing World Bioethics (2025): 1–7, 10.1111/dewb.70001. PMC12980560 40679088

[77] A. Edinger , D. Valdez , E. Walsh‐Buhi , et al., “Misinformation and Public Health Messaging in the Early Stages of the Mpox Outbreak: Mapping the Twitter Narrative With Deep Learning,” Journal of Medical Internet Research [Electronic Resource] 25 (2023): 1–11, 10.2196/43841. PMC10282910 37163694

[78] A. Edinger , D. Valdez , E. Walsh‐Buhi , et al., “Misinformation and Public Health Messaging in the Early Stages of the Mpox Outbreak: Mapping the Twitter Narrative With Deep Learning,” Journal of Medical Internet Research (2023): 25, e43841. 37163694 10.2196/43841 PMC10282910

[79] Z. Linfeng , R. Bhuvanagiri , B. Gonzales , K. Sharp , and D. Murthy , “A Dashboard Approach to Monitoring Mpox‐Related Discourse and Misinformation on Social Media,” preprint, arxiv, May 26, 2025.

[80] M. Edward , O. Shadrack , S. T. Engmann , and A. Jubilate , “Global Mpox Transmission and Stigma: Addressing Barriers in Affected Communities,” Discover Public Health 22, no. 1 (2025): 4–7, 10.1186/s12982-025-00470-4.

[81] A. Acharya , N. Kumar , K. Singh , and S. N. Byrareddy , “Mpox in MSM: Tackling Stigma, Minimizing Risk Factors, Exploring Pathogenesis, and Treatment Approaches,” Biomedical Journal 48, no. 1 (2025): 100746, 10.1016/j.bj.2024.100746. 38734408 PMC11751411

[82] C. Manirabarusha , Stigma Adds to Burundi's Challenges in Mpox Fight (Reuters, 2024).

[83] S. B. Mambo , G. M. E. Nja , U. O. Bunu , et al., “Promoting Risk Communication and Community Engagement During Mpox Outbreak in Fragile Conflict Zones of Eastern DRC,” One Health 20 (2025): 101012, 10.1016/j.onehlt.2025.101012. 40160936 PMC11951864

[84] V. S. Mithi , “Mpox in Africa: Funding Cuts and Delayed Global Actions Fuelling New Epicentres,” Lancet Global Health 13, no. 9 (2025): e1512–e1513, 10.1016/S2214-109X(25)00241-4. 40651484

